# DEMENTIA LIFE EXPECTANCIES: NEW KNOWLEDGE AND CONSIDERATIONS FROM THE HEALTH AND RETIREMENT STUDY

**DOI:** 10.1093/geroni/igac059.1349

**Published:** 2022-12-20

**Authors:** Marc Garcia, Wassim Tarraf, Chi-Tsun Chiu, Joseph Saenz, Adriana Reyes

**Affiliations:** Syracuse University, Syracuse, New York, United States; Wayne State University, Detroit, Michigan, United States; Institute of European and American Studies, Taipei City, New Taipei, Taiwan (Republic of China); University of Southern California, Los Angeles, California, United States; Cornell University, Ithaca, New York, United States

## Abstract

Alzheimer's disease and related dementias (ADRD) are a growing public health crisis. Estimates on the prevalence and incidence of ADRD across and within population-based studies have varied in part due to competing measures to assess dementia status. Disentangling these inconsistencies is crucial for understanding dementia disparities among racial/ethnic, and nativity groups among older adults. Based on the Health and Retirement Study we examined across (Whites, Blacks) and within-group differences (US- and non-US-born Latinos) in estimates of dementia life expectancy, using four competing algorithmic techniques (i.e., the Langa-Weir, Expert, Hurd, and Lasso) for the classification of dementia ascertainment. Estimates of dementia life expectancy across algorithms largely point to dementia disparities in the prevalence of the disease across racial/ethnic, and nativity groups, regardless of the algorithmic technique utilized. Elucidating algorithms that can be utilized with different racial/ethnic groups may reduce bias in dementia assessment in the future.

